# Revisiting health systems to integrate perinatal mental health into maternal and child health services: perspectives from research, policy and implementation

**DOI:** 10.1136/bmjgh-2024-015820

**Published:** 2024-12-11

**Authors:** Anteneh Asefa, Charlotte Hanlon, Bruno Marchal, Caroline Homer, Samson Gebremedhin, Özge Tunçalp, Nandini Sarkar, Alexandre Delamou, Shanon McNab, Lenka Beňová

**Affiliations:** 1 Department of Public Health, Institute of Tropical Medicine, Antwerp, Belgium; 2 Division of Psychiatry, Centre for Clinical Brain Sciences, University of Edinburgh, Edinburgh, Scotland, UK; 3 Department of Psychiatry, School of Medicine, College of Health Sciences, Addis Ababa University, Addis Ababa, Ethiopia; 4 Center for Innovative Drug Development and Therapeutic Trials for Africa (CDT-Africa), College of Health Sciences, Addis Ababa University, Addis Ababa, Ethiopia; 5 Maternal, Child and Adolescent Health Program, Burnet Institute, Melbourne, Victoria, Australia; 6 School of Public Health, Addis Ababa University, Addis Ababa, Ethiopia; 7 UNDP/UNFPA/UNICEF/WHO/World Bank Special Programme of Research, Development and Research Training in Human Reproduction (HRP), Department of Sexual and Reproductive Health and Research, World Health Organization, Geneva, Switzerland; 8 Centre National de Formation et de Recherche en Santé Rurale, Maférinyah, Forécariah, Guinea; 9 Africa Center of Excellence (CEA-PCMT), Gamal Abdel Nasser University of Conakry, Conakry, Guinea; 10 MOMENTUM Country and Global Leadership, Jhpiego, Baltimore, Maryland, USA

**Keywords:** health systems, health services research, health policy, mental health & psychiatry, public Health

**Summary box**

- There is a growing call for actionable recommendations to promote perinatal mental health and to prevent and treat perinatal mental health conditions, particularly in Africa.
- The design and implementation of perinatal mental health promotion and prevention interventions should be (re)imagined based on the tenets of woman-centred approaches that recognise the unique needs and expectations of women.
- Caring for health workers in general and their mental health in particular should be an integral part of perinatal mental health interventions.
- There are proven and cost-effective interventions which can avert the burden of poor perinatal mental health in low-resource settings, if supported by political commitment, context-adapted policies and strategies, adequate investment, intersectoral collaboration, community engagement, and ongoing research.

## Introduction

 Given the concerning prevalence and large-scale impact of poor perinatal mental health worldwide, there is a growing call for actionable recommendations to promote perinatal mental health and to prevent and treat perinatal mental health conditions. In this commentary, we reflect on existing evidence on the (potential) contributors to perinatal mental health conditions and put forward research, policy and implementation considerations for advancing perinatal mental health, with a focus on the African region.

### The burden of poor perinatal mental health

Globally, the burden of common perinatal mental health conditions—mainly anxiety disorders and depression—is substantial, with individual-level to societal impacts, which can impede achievement of the Sustainable Development Goals.^1^ Nearly one in five (19.8%) women in low- and middle-income countries experience common mental disorders.^2^ While perinatal depression—depression that occurs during pregnancy or after childbirth—contributes an important share of perinatal mental health conditions, there is a significant unmet need for measures to address it.^3 4^ Current evidence suggests that women’s negative experiences of care during childbirth may contribute to postpartum depression.^5^ There is a growing body of evidence for higher levels of postpartum depression among women and adolescents who have experienced mistreatment during facility-based childbirth (also known as disrespect and abuse or obstetric violence).^6 7^

### Why Africa?

Africa has the highest rates of adolescent pregnancy, mistreatment during childbirth, maternal morbidity and mortality and perinatal depression (17% compared with 10% globally).^8^,^11^ These challenges are inextricably linked and share deep-rooted drivers,^3 12^ meaning they must be addressed through context-appropriate, coordinated and multisectoral interventions. Such interventions should prioritise tackling the contributors to poor access to high-quality maternal, sexual and reproductive health services. Low coverage and quality of routine postnatal care (48%)^13^ and lack of integration of mental health services into the continuum of maternal healthcare spanning from the preconception period to the postpartum period^14^ contribute to low detection and treatment of perinatal depression in African countries.^15^ This in turn leads to a host of individual-, family and societal-level problems. For women, this can include difficulty functioning, suicidal behaviour, poorer self-care, substance use, less optimal engagement with maternal care services, and poorer pregnancy and obstetric outcomes.^4^ Perinatal depression in the woman is also associated with increased mental ill-health in her partner, compromised child health, growth and development and lost productivity and poverty.^4^ A recent report from the World Economic Forum highlighted that closing the health gap in depressive symptoms among women has the potential to add at least US$100 billion to the global economy.^16^ Stigma and discrimination can exacerbate the severity of perinatal depression and be a potent barrier to accessing services.

### Contributors to poor perinatal mental health: evidence gaps and research priorities

There exists an extensive evidence-base on risk factors for postpartum depression (box 1A). An increasingly recognised factor in poor perinatal mental health is the mistreatment (also called ‘disrespect and abuse’ or ‘obstetric violence’) of women during childbirth in health facilities (box 1B).

Box 1 **Poor perinatal mental health and mistreatment of women during childbirth**

**A. Contributors to poor perinatal mental health**

These include, among others, intimate partner violence or marital discord, economic stress and other life stressors, lack of social support, unplanned pregnancy, poor perinatal outcomes, and pregnancy and childbirth complications.^37^ Moreover, misconceptions and low awareness of perinatal depression among women, families and health workers, contextual conditions and stressors such as armed conflict and migration, climate change, pandemics such as the COVID-19 crisis and political and economic instability, exacerbate its prevalence and severity.^1738^,^41^

**B. Mistreatment of women during childbirth in health facilities and its role in poor perinatal mental health**

**Figure 1:**
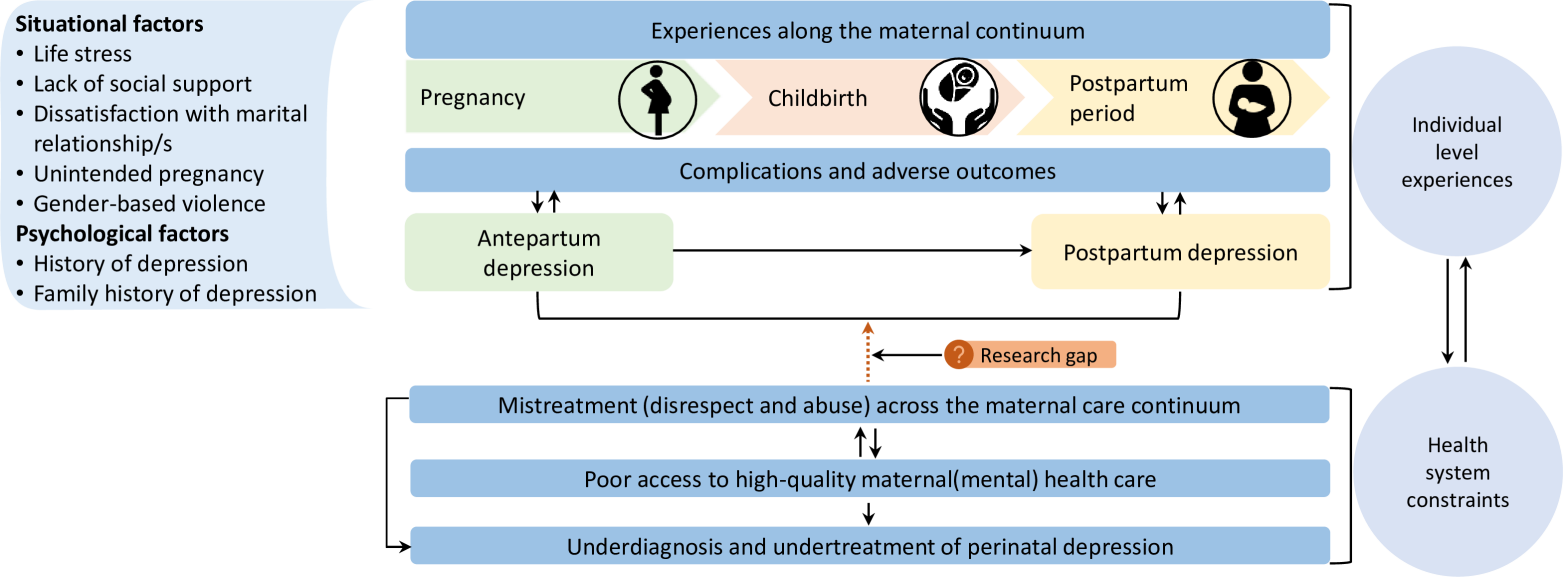
Perinatal depression, risk factors and consequences.

Mistreatment may take various forms, such as physical, sexual and verbal abuse; non-dignified care; compromised standards of care; poor rapport between health workers and women and their families and health system bottlenecks, including conditions that compromise labour support and companionship.^9^ Mistreatment of women is a key issue that influences the experience and quality of care, as well as future care-seeking, including for psychosocial problems and mental health conditions.^42 43^ Evidence from several low- and middle-income countries, including those in Africa, shows a worryingly high prevalence (28%–83%) of mistreatment of women during childbirth in health facilities.^9^ Studies from Brazil,^7^ and Ghana, Guinea, Myanmar and Nigeria^6^ reported a significantly higher prevalence of postpartum depression among women who experienced mistreatment during childbirth in health facilities. Recently, a high-level global technical consultation highlighted the urgent need to generate better evidence on the links between the mistreatment of women during childbirth in health facilities and perinatal mental health.^34^ This requires examining the role of mistreatment in a potentially complex causal chain between perinatal depression and its various risk factors or consequences using robust epidemiological and health systems research approaches (figure 1). Such research should account for the role of determinants of both mistreatment and perinatal depression, as well as follow women across the maternal health continuum—from pregnancy to the late postpartum period and beyond. The benefits of longitudinal studies measuring symptoms of perinatal depression, its risk factors and experiences of mistreatment have a dual benefit. First, they provide a better estimate of the role of mistreatment in postpartum depression and women’s well-being more generally. Second, they capture changes in depressive symptoms over the maternal continuum. To enhance the rigour of such longitudinal studies, researchers should embed qualitative approaches that explore the lived experiences of women affected by perinatal depression and involve them in the design and conduct of studies. It is especially pressing to explore the experiences of adolescent women as there is a critical evidence gap in perinatal mental health for this key population.^44^

### Policy and implementation(research) perspectives

#### Towards women-centred and respectful perinatal mental health systems

Strengthening perinatal mental health promotion, prevention and treatment services in Africa is compounded by various interconnected challenges. These include underinvestment in health systems, staff shortages and heavy reliance on task-shifted maternal (mental) health services, lack of staff training in screening/treatment of perinatal depression, diverse sociocultural expectations of care, normalisation of mistreatment of women during childbirth as well as societal factors, including high levels of gender-based violence, and low awareness and stigmatisation of people with mental conditions.^17^,^20^ Therefore, the design and implementation of perinatal mental health promotion and prevention interventions should be (re)imagined based on the tenets of woman-centred approaches that recognise the unique needs and expectations of women. Engaging women and communities in co-designing and implementation of perinatal mental health interventions is essential to building their trust in available prevention and treatment and improving their retention in care.

Formative research should focus on critically examining the ecosystem of maternal health and understanding the needs of pregnant and postpartum women and their families. In the process, strengthening social accountability mechanisms is pivotal not to leave behind adolescent and young women, ethnic minorities, migrants, women living with HIV, economically deprived families and women in humanitarian settings.^18^ Likewise, an integral component in the whole process of promoting beneficial and relevant perinatal mental health services is providing adequate answers to the question ‘Do women and communities trust our health services?’ Thus, it is critical to examine and tackle the complex drivers of health workers’ and users’ (mis)trust in perinatal mental health services and the overarching health system.^21^ An important contribution would be to build connections with religious and traditional healers, as they often hold significant roles within the community in relation to perceptions about mental health conditions and their treatment.^22^

#### Integrated perinatal care provision

The integration of perinatal mental health interventions into maternal health services at macro-level, meso-level and micro-level of health systems is an essential requirement towards woman-centred healthcare systems. A recent guide launched by WHO identifies key aspects required for integrated physical and mental perinatal care to ensure and promote the mental well-being of perinatal women in ways that are socio-culturally and contextually appropriate (box 2).^15^

Box 2 **WHO’s stepped-care approach to the provision of perinatal mental healthcare^15^**

1. Promotion of good mental health and well-being for all women through multidimensional interventions, including equipping women with information and skills to maximise their own mental health, stress management, social support, life skills education and screening for mental health conditions.
2. Preventive interventions for vulnerable women who have time-limited mental health concerns that affect their functioning.
3. Treatment of mental health conditions with mild-to-moderate symptoms and referral to specialist care for women whose symptoms do not improve.
4. Treatment of mental health conditions with moderate-to-serious symptoms by mental health specialists.

We argue that it is crucial to first strengthen the capacity within maternal care to provide basic perinatal mental health services. This could begin with incorporating mental health modules in in-service training and mentorship of frontline health workers providing maternal and child health services. Resources include the WHO mental health Gap Action Programme, now implemented in >100 countries^23^ and the Practical Approach to Care Kit, which integrates woman-centred psychosocial approaches into every primary care-based maternal care consultation along the continuum of care using a problem-focused approach.^24^ Strengthening effective supervision, consultation and referral systems for perinatal mental healthcare to tertiary care facilities, using the stepped-care approach,^15^ is essential, particularly in contexts where available mental health services are concentrated in hospitals. On the other hand, leveraging resources to forge perinatal mental health interventions at the community level and involving women who have experienced perinatal depression in the development and improvement of services is vital to improve early prevention, identification and early referral of perinatal mental health conditions.^25^ Incorporation of perinatal mental health indicators into existing maternal health monitoring and evaluation systems is the foundation for strengthening a culture of continuous quality improvement through generating evidence for learning health systems and redesigning local maternal health policy and implementation.^26^ Without these structural shifts in health systems, any investment in maternal care will fall short of ensuring the accessibility of truly holistic and woman-centred care across the maternal continuum of care. Embedding implementation research in integration and quality improvement initiatives is an excellent opportunity to contribute to the (co)production of knowledge that can be applied in the local context to promote perinatal mental health.^27^ In tandem, the meaningful engagement of frontline health workers in such implementation research will have far-reaching benefits in enhancing ownership and sustainability of interventions.

#### A midwifery-led model: a promising model for improving facility-based perinatal mental health in Africa?

A systematic review reported that strengthening midwifery continuity of care contributes to improved perinatal mental health in high-income settings.^28^ In low-resource settings, there is growing evidence that midwifery continuity of care models that have respectful maternity care at their heart improve the uptake and quality of maternal health services.^29^ However, there is little or no evidence of their impact on improving perinatal mental health. As midwives and nurses provide most of the care along the pregnancy, childbirth and the postpartum continuum in most countries in Africa, it is imperative to explore how to integrate interventions to improve perinatal mental health into such continuity of care models. We propose this should be approached first from a bottom-up perspective by testing the effectiveness of context-appropriate models, and gradually scaling these up through a top-down political and administrative commitment. There is a need to re-think the training of midwives and general health workers involved in maternal care so that they are all equipped with the necessary skills for compassionate communication, building rapport and providing basic psychosocial support. Adult learning-based approaches show promise.^30^ The onus is not on midwives alone as current systems mitigate against woman-centred care. When midwives are overwhelmed due to inadequately resourced health systems and extensive social adversity, their capacity to deliver woman-centred care is undermined. Innovative approaches for integrating interventions to address the social determinants of poor perinatal mental health from a multidisciplinary perspective, for example, cash transfers and interventions to reduce gender-based violence, need testing at larger scale.^31 32^ Alongside this, in light of the very high levels of burnout among health workers in under-resourced health systems,^33^ we underscore that caring for health workers in general and their mental health in particular should be an integral part of perinatal mental health interventions.

## Conclusions

Poor perinatal mental health remains a neglected public health challenge in Africa, despite recognised intergenerational consequences and negative impact on maternal and newborn health.^14^ There is an urgent need for more research and resources to inform and implement policy on universal care for perinatal mental health.^34^ The cost of inaction to prevent and treat peripartum depression poses far-reaching consequences at the individual, family and national levels^35^ on top of derailing efforts to achieve maternal and mental health targets of the Sustainable Development Goals.^36^ Despite the challenges, there are proven and cost-effective interventions that can avert the burden of poor perinatal mental health in low-resource settings,^20^ if supported by political support, context-adapted policies and strategies, adequate investment, intersectoral collaboration, women’s and community engagement and ongoing research. One such approach—valuable in itself—is improving respectful care during pregnancy, childbirth and the postpartum period. Now is the time to explore and strengthen synergies between innovative interventions to improve respectful maternity care and perinatal mental health services in the region and globally.

## Data Availability

There are no data in this work.
